# Diabetes

**Published:** 1890-09-20

**Authors:** 


### DIABETES.

Dr. Pavy's article "On the Principles of the Treatment of Diabetes Mellitus," read before the International Medical Congress at Berlin, demands attention. It is with the carbo-hydrate constituents of the food, Dr. Pavy asserts, that faulty action lies. These, instead of being converted and utilised, are eliminated as carbo-hydrates. The carbo-hydrate in the form of sugar reaches the general circulation in a manner it ought not to do. The condition of the urine as regards sugar affords an index of the state of the blood, for the sugar eliminated is not *formed* in the kidney. We have to deal with a failure of power, to arrest the passage of carbo-hydrates through the liver. Carbo-hydrates reach the circulation, are eliminated by the kidneys, and are lost. The disease thus involves a sacrifice of material which ought to be turned to account. If the vital chemistry of digestion could be put right, the disease would be removed. But it is not generally possible to restore the transformative or assimilative power, and the only way of arriving at what is wanted is to stop the introduction into the system of those alimentary principles which cannot be properly disposed of. As long as passage of sugar through the system is prevented no harm takes place. What in reality inflicts harm is the altered constitution of the blood occasioned by the presence in it of sugar. Nutrition is not properly carried on. Dr. Pavy, however, does not tell us how sugar in the system prevents nutrition being carried on properly. But he says as a consequence the general powers become sapped and exhausted. Although Dr. Pavy tells us the disease involves "a sacrifice of material which ought to be turned to account," he also says: "If it were only a question of waste of the carbo-hydrate principles of food, there would be no reason against their being taken and allowed to run off. Provided a sufficient amount of other alimentary principles were consumed to meet the requirements of life, no particular harm need arise from the sacrifice of carbo-hydrates." Of this, however, we do not feel sure. For the maintenance of health, if not the requirements of life demand a certain assimilation of carbo-hydrate principles. But Dr. Pavy considers that the first consideration is to control by dietetic means the passage of sugar through the system, while a point to be aimed at is the restoration of the assimilative power over the carbo-hydrate elements of food. What most conduces to this desired restoration is the maintenance of a normal state of the system by keeping it free from the passage of sugar. Opium and its derivatives, codeine and morphine, are the medicinal agents which assist in the restoration of assimilative power. Now, with all due deference to Dr. Pavy, it is many years since the late Dr. J. Wright asserted that diabetes is not a disease of the kidneys. He attributed diabetes to defect of digestion. But Dr. Pavy and some others have gone a step further, and attributed diabetes solely to defective hepatic action, being chiefly guided to this conclusion by finding a large preponderance of sugar in the blood of the portal vein of animals after the ingestion of food containing much carbo-hydrate material. But in our opinion the vital process of digestion is so complex that we cannot yet discriminate with certainty what organ is in fault when digestion is imperfect. Dr. Saundby has recently published his "Bradshaw lecture on the morbid anatomy of diabetes mellitus" (*Brit. Med. Journal*, August 23). Dr. Saundby says that great interest attaches to the liver because of its physiological relations to sugar formation. In Dr. Saundby's experience, fatty degeneration is very common, and a certain degree of interstitial hepatitis is frequently present. There is not, however, any special condition which may be regarded as characteristic of diabetes. Other observers have stated that the liver in diabetes is usually healthy, and sometimes presenting "even a more than usually healthy appearance." Dr. Saundby further mentions that, in his opinion, the wasting of the pancreas is the most important lesion in diabetes. Out of fifteen cases, it was abnormal in eleven. Saundby agrees with Lancereaux that the pancreas will be found atrophied in all typical cases of wasting diabetes. Then there is the experience of Bull, of New York, whose patient died of diabetes after extirpation of the pancreas. The liver changes, Dr. Saundby thinks, are altogether secondary. There is, unfortunately, the fact that we do not fully understand the part that each organ takes in digestion and assimilation. And Dr. Saundby's elaborate essay on the morbid anatomy of diabetes does not assist us much in arriving at a conclusion as to the organ specially defective in this disease.

